# Parametric study of 3D printed microneedle (MN) holders for interstitial fluid (ISF) extraction

**DOI:** 10.1007/s00542-020-04758-0

**Published:** 2020-02-01

**Authors:** Robert M. Taylor, Dilendra Maharjan, Fernando Moreu, Justin T. Baca

**Affiliations:** 1 Department of Emergency Medicine, The University of New Mexico, MSC11 6025, Albuquerque, NM 87131 USA; 2 Department of Civil, Construction and Environmental Engineering, Courtesy Appointment, The University of New Mexico, Centennial Engineering Center 3056, MSC01 1070, Albuquerque, NM 87131 USA; 3 Department of Electrical and Computer Engineering, Courtesy Appointment, The University of New Mexico, Centennial Engineering Center 3056, MSC01 1070, Albuquerque, NM 87131 USA; 4 Department of Mechanical Engineering, The University of New Mexico, Centennial Engineering Center 3056, MSC01 1070, Albuquerque, NM 87131 USA

## Abstract

The need for novel, minimally invasive diagnostic, prognostic, and therapeutic biomedical devices has garnered increased interest in recent years. Microneedle (MN) technology has stood out as a promising new method for drug delivery, as well as extraction of interstitial fluid (ISF). ISF comprises a large portion of the extracellular fluid in living organisms yet remains inadequately characterized for clinical applications. Current MN research has focused on the fabrication of needles with different materials like silicone, carbon, and metals. However, little effort has been put forth into improving MN holders and patches that can be used with low cost MNs, which could effectively change how MNs are attached to the human body. Here, we describe different 3D-printed MN holders, printed using an MJP Pro 2500 3D printer, and compare the ISF extraction efficiencies in CD Hairless rats. We varied design parameters that may affect the skin-holder interface, such as throat thickness, tip curvature, and throat diameter. MN arrays, with insertion depths of 1500 μm, had extraction efficiencies of 0.44 ± 0.35, 0.85 ± 0.64, 0.32 ± 0.21, or 0.44 ± 0.46 µl/min when designed with flat, concave, convex, or bevel profile geometries, respectively. Our results suggest ISF extraction is influenced by MN holder design parameters and that a concave tip design is optimal for extracting ISF from animals. The future direction of this research aims to enable a paradigm in MN design that maximizes its efficiency and engineering performance in terms of volume, pressure, and wearability, thereby automatizing usage and reducing patient intervention to ultimately benefit remote telemedicine.

## Introduction

Advancement in modern drug delivery methods have unlocked numerous opportunities in the treatment and diagnosis of diseases (Chandel et al. 2019; Jain 2014; Li et al. 2017; Lim et al. 2018; Pandey et al. 2019). Ingestion, inhalation, absorption, and intravenous injection are the most common methods of delivering drugs (Jain 2014). Transdermal drug delivery methods have also been used when non-invasive methods are preferred (Akhtar et al. 2020; Gonnelli and McAllister 2011). However, non-needle based methods may not be suitable for larger molecular weight compounds like vaccines (Gonnelli and McAllister 2011). On the other hand, typical injections with hypodermic needles can cause pain and discomfort for patients with possible damage to veins and bruising. In this context, microneedles (MN) have gained significant research interest for innovative drug delivery and monitoring methods (Akhtar et al. 2020; Kiang et al. 2017; Rzhevskiy et al. 2018).

MN technology has also recently been utilized for interstitial fluid (ISF) extraction and analysis. ISF comprises a large portion of the extracellular volume in living organisms (Cengiz and Tamborlane 2009). However, until recently, there has been a lack of adequate technology for the extraction of sufficient volumes of ISF for downstream analysis. Thus, ISF has been inadequately characterized for biomedical or clinical applications. We recently devised MN arrays (MAs, which includes microneedles extending from a 3D-printed holder) that are able to extract upwards of 20 μL and 60 μL of ISF in 1 h from both humans and rats, respectively (Miller et al. 2018; Taylor et al. 2018; Tran et al. 2018). These volumes of ISF are adequate for downstream analysis such as transcriptomics (Miller et al. 2018), proteomics (Tran et al. 2018), and metabolomics (Miller et al. 2018; Taylor et al. 2018).

MAs are designed to penetrate up to the intracutaneous layer to avoid blood extraction (Gartstein et al. 2002; Kiang et al. 2017) and pain. With this technique, drug delivery can be achieved with minimal pain and minimal damage to skin and veins. This technique has also been experimentally verified to assist in extraction of ISF from the dermis layer (Miller et al. 2018; Taylor et al. 2018; Tran et al. 2018; Wang et al. 2005). MNs have also been used for drug and vaccine delivery (He et al. 2019; Kim et al. 2012). Since MNs are minimally-invasive, compared to conventional injecting devices, they are generally easy to use and less painful for human use (Gill et al. 2008; Haq et al. 2009; Wang et al. 2005). This demonstrates the great market potential for MNs as prognostic, diagnostic, and drug delivery methods (Lee et al. 2020).

Extensive research has been conducted into the structural design, materials selection, and fabrication of MNs/MAs (Kim et al. 2012; Chaudhri et al. 2010). Research has also shown that the tip design of a MN may affect the efficiency of fluid extraction (Ma and Wu 2017). ISF flow and skin biomechanics have also been studied in relation to various MN extraction techniques such as hydrogel and hollow microneedles (Samant and Prausnitz 2018). Other parameters to consider for the MN design are size, diameter, insertion depths, and types of surface coatings used on the MN (Kim et al. 2012). However, a limited amount of literature can be found for the design of the MN holder itself, which houses the MN and interfaces with the skin region in proximity to the extraction site. Table 1 shows the different MN holder design types, available to date, used to extract ISF or to apply a drug. It is important to note that fluid extraction from the deep dermis layer not only depends on the MNs but also on the MN holders used to house the MNs and to apply them to the tissue. One of the important observations when using different types of MAs is that extraction may also be influenced by the pressure exerted by the holder during the fluid extraction process (Miller et al. 2018; Samant and Prausnitz 2018). It would be of interest to users to enable extraction of ISF with a reduced amount of pressure. One advantage of designing an MA for ISF extraction without pressure includes the ease of patient use, so that the individual patient can apply the MA without a prescribed pressure input. Another benefit is that the system becomes automatic. Previous research has shown that 3D printed MN holders are able to aid in the collection of the ISF using capillary tubes without applying any mechanical suction to the skin (Taylor et al. 2018). Results demonstrated that volume can be obtained without pressure under certain design types. However, to date, there is little knowledge about the design of MNs/MAs and the volume of the ISF extracted.

**Table 1:**
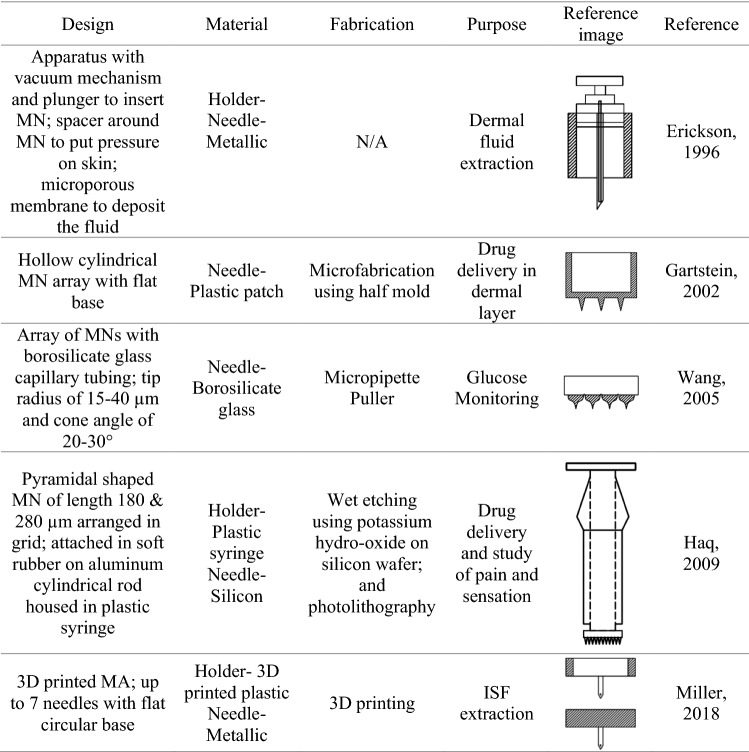
MN holder design types

Here, we present the design, fabrication, and testing of different models of MN holders in order to improve the maximum amount of ISF extracted. More specifically, we conducted a parametric study of 3D printed MN holders for ISF extraction. Such holders can be used in conjunction with several MNs, thus forming the MA. Overall, we developed 8 different 3D printed MAs with design parameters that were altered to achieve optimum ISF extraction rates. The following sections of this paper outline the various methods and materials.

## Fabrication design, methods, and testing procedures

This section covers the conceptual development of the different topologies proposed. The design optimizations explore various interfaces that will enhance or augment the performance of the MN, under assumptions of similar pressure with limited supervision. The fabrication of such devices is also described in order to emphasize the quick manufacturing aspect of the proposed prototype. Finally, the authors outline the basic testing protocols informing the results, and their relevance towards the validation of the new MN.

### MN holder designs

**Fig. 1:**
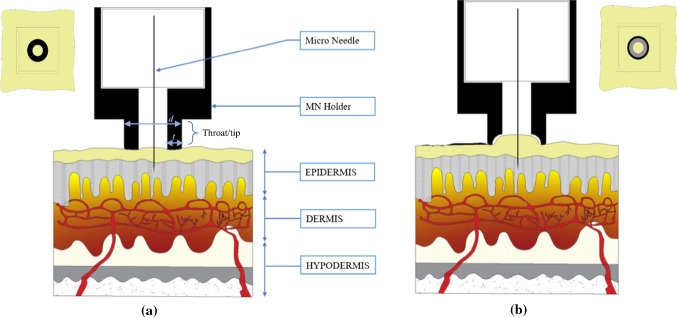
Flat (**a**) and Concave (**b**) tips of MN holders interfacing with human skin. Parameters include throat thickness *t* and throat diameter *d*

We primarily focused on the throat/tip region of the MN holders. Four different designs were developed, each having a unique interface with the skin. Such interfaces differed due to the geometric profile of the throat/tip and the contact surface area of the tip. Figure 1 illustrates two of the MN holder types: flat and concave when in contact with skin. Given that each holder is applied to the skin in the same manner, the contact surface of the profile geometry determines the pressure applied to the skin. The flat tip has more contact surface hence, less net pressure compared to a concave tip. The holders were designed in such a manner as to allow 1000 μm or 1500 μm of the MN to be inserted into the skin. However, the change in skin surface geometry due to applied pressure may vary this depth, which may cause variations in the ISF extraction volume. The MN holders used are the result of rapid prototyping without complex manufacturing processes. Hence, these designs are meant to be portable and recreated without much technical expertise. The MN holders utilize commercially available MNs, such as BD Ultra-Fine Pen Needles that are pre-sterilized and can be installed in the 3D-printed MN holders without requirement of direct supervision from medical practitioners. We believe the simple design for producing these holders would allow for the democratization for off-site diagnosis using MAs.

### 3D printing of MN holders

**Fig. 2:**
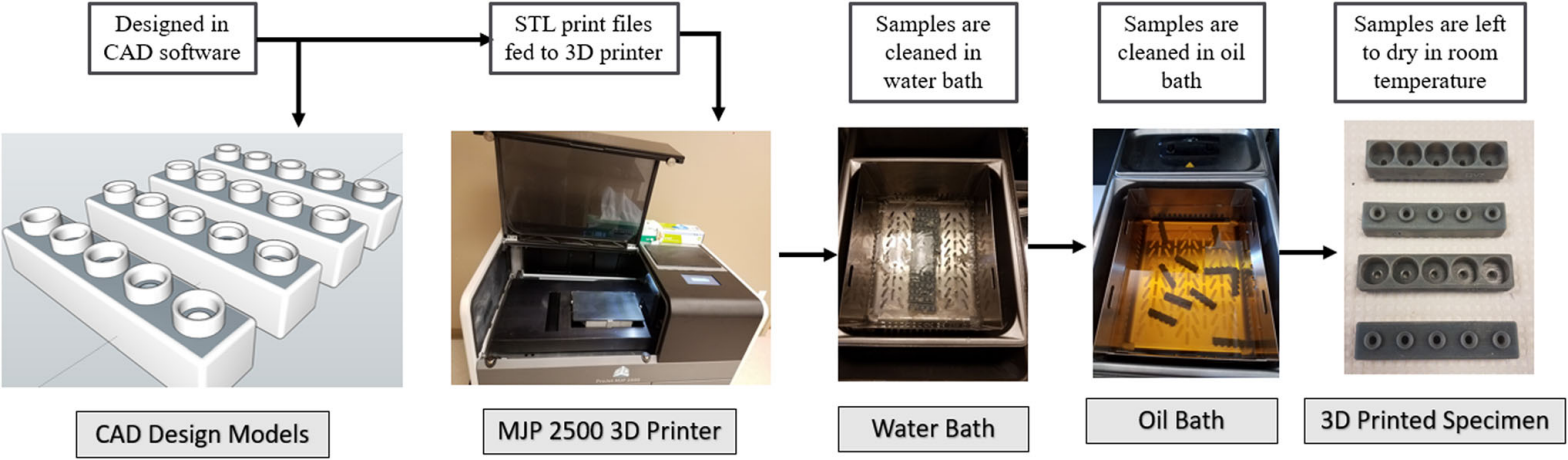
MN holder design and print workflow

SketchUp computer aided design (CAD) software was used for designing the MN holders. The software was used to specify dimensions up to sub millimeter precision and designs were exported to an object file (.stl) that could be read in the 3D printer’s user interface, called 3D Sprint (3D Systems, Inc., Rock Hill, SC, USA). The resolution of printer varies depending on the orientation of the specimen placed on the build plate. The print resolution is highest in the z direction which is 0.02 mm. MN holders were 3D printed using a commercially available ProJet MJP 2500 printer (3D Systems, Inc.) using a VisiJet^®^ M2R-GRY build material and a VisiJet^®^ M2 SUP support material, both from 3D Systems. The 3D printer uses support material to provide adhesion to the main print material and makes the printing mechanism more agile, hence able to print more complex shapes. After a layer of material is deposited on the printing bed, it is exposed to a flash of ultraviolet rays to cure the material. After printing, the MN holders were placed at −20 °C for 5 min to release the printed holders from the base plate. MN holders were then placed in a steam bath for 15 min to remove the wax support material and subsequently placed in a hot oil bath for another 15 min to remove all traces of the wax support. MN holders were then cleaned using hot tap water and soap and left at room temperature to dry. Figure 2 details the design, printing, and post-processing steps.

### MA assembly and animal testing

The animal care and use program of the University Of New Mexico (UNM) is accredited by AAALAC International and the UNM’s animal care and use committee approved all experiments. CD hairless, Crl:CD-Prss8hr, rats (Charles River Laboratories, Wilmington, MA) were used for the studies. ISF was extracted using our previously published methods (Tran et al. 2018). Briefly, animals were anesthetized using 2% isoflurane, and MAs were applied to extract ISF. Ultra-fine Nano PEN needles (BD, Franklin Lakes, NJ) were placed into the 3D-printed MN holders to form the MA. Each needle was attached to a 1–5 µl calibrated pipet capillary tube (Drummond Scientific Co., Broomall, PA). The array assembly was then pressed into the dermal tissue of the rats and held in place for exactly 2.0 min per extraction. The volume of ISF extracted in each needle and the total ISF extracted per MA was recorded for each extraction. All animals had a terminal cardiac puncture performed at the conclusion of the experiments.

## Results and discussion

### MA design and printing

**Fig. 3:**
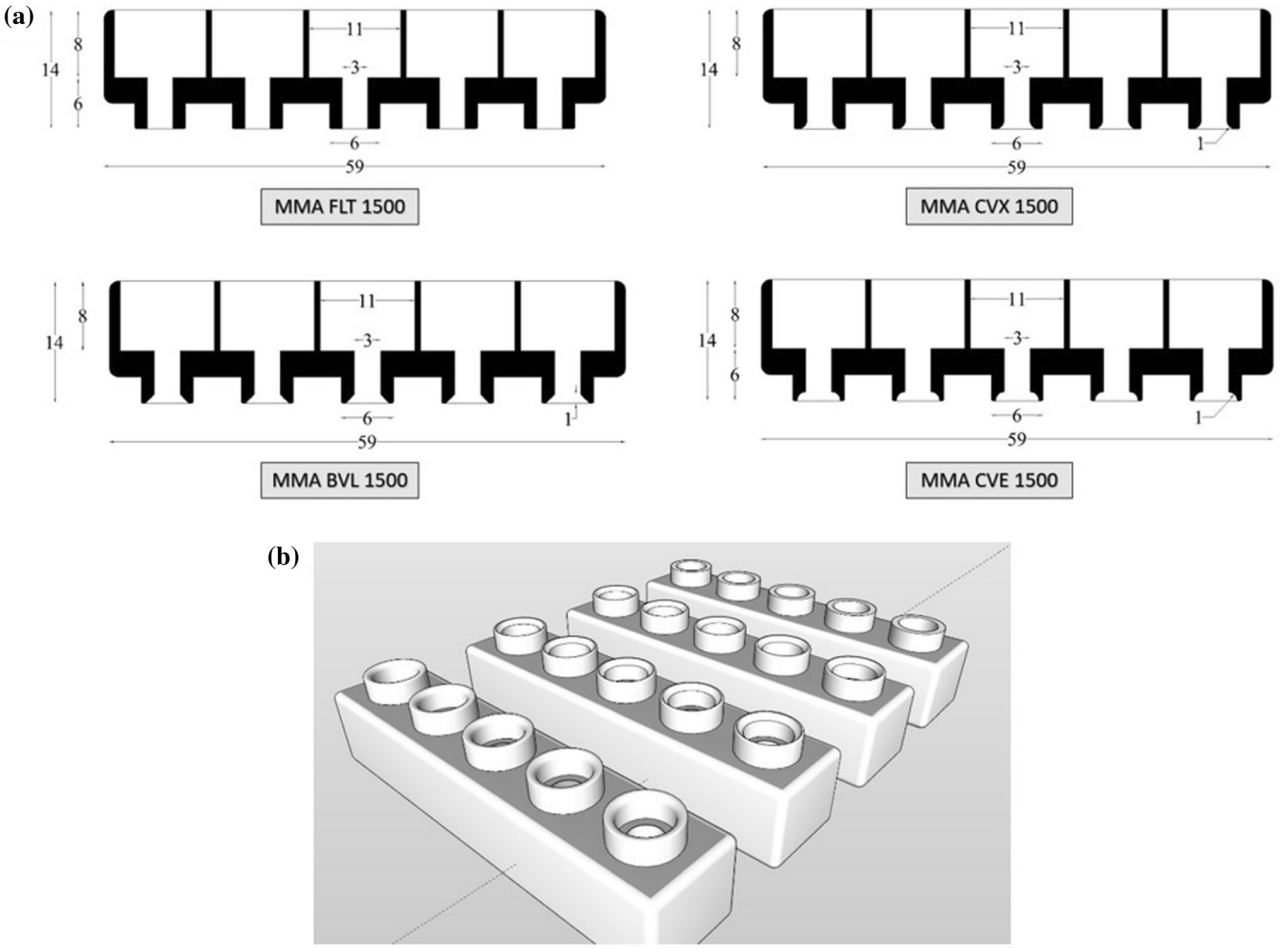
**a** CAD drawing of four specimens. Dimensions are shown in mm, **b** 3D designs of four specimens exported from SketchUp to printer

We investigated the effect of different throat/tip geometry profiles for the holders. Figure 3 shows the four MA designs that were tested: flat, convex, concave, and beveled. The flat prototype (Fig. 3a) is considered the base design, and all other designs include a modification of the throat/tip of this base design. Table 2 shows the tip parameters that were modified to create different versions of the design. Additionally, each MA prototype was designed and tested with both a 1000 μm and 1500 μm needle insertion length.

**Table 2:** MA throat/tip parameters

Design ID	Profile geometry	Insertion depth (µm)	Tip curvature (mm)
FLT	Flat	1000 and 1500	Flat profile w/o curvature
CVE	Concave	1000 and 1500	1 mm radius rounded concave
BLV	Bevel	1000 and 1500	1 mm bevel offset from edge
CVX	Convex	1000 and 1500	1 mm radius rounded convex

### MA prototype testing in CD hairless rats

**Fig. 4:**
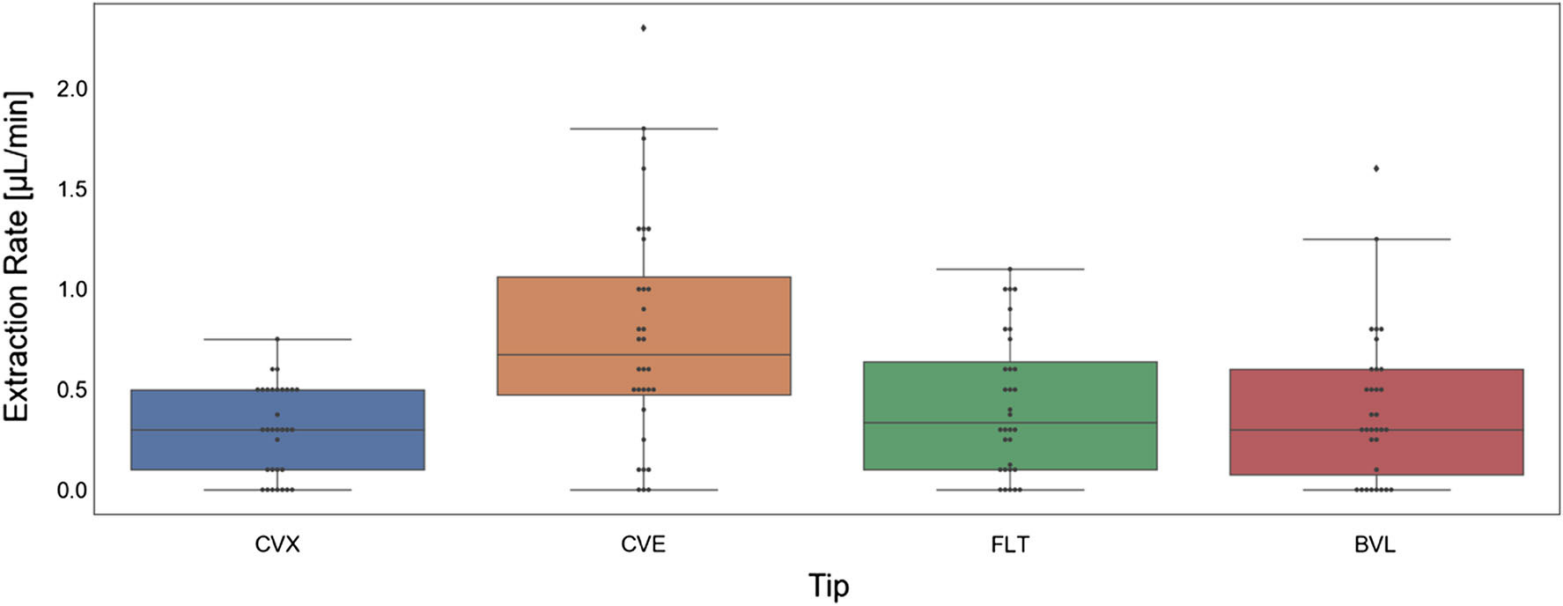
MA prototype extraction rates in CD hairless rats (*n* = 32 for each prototype)

**Fig. 5:**
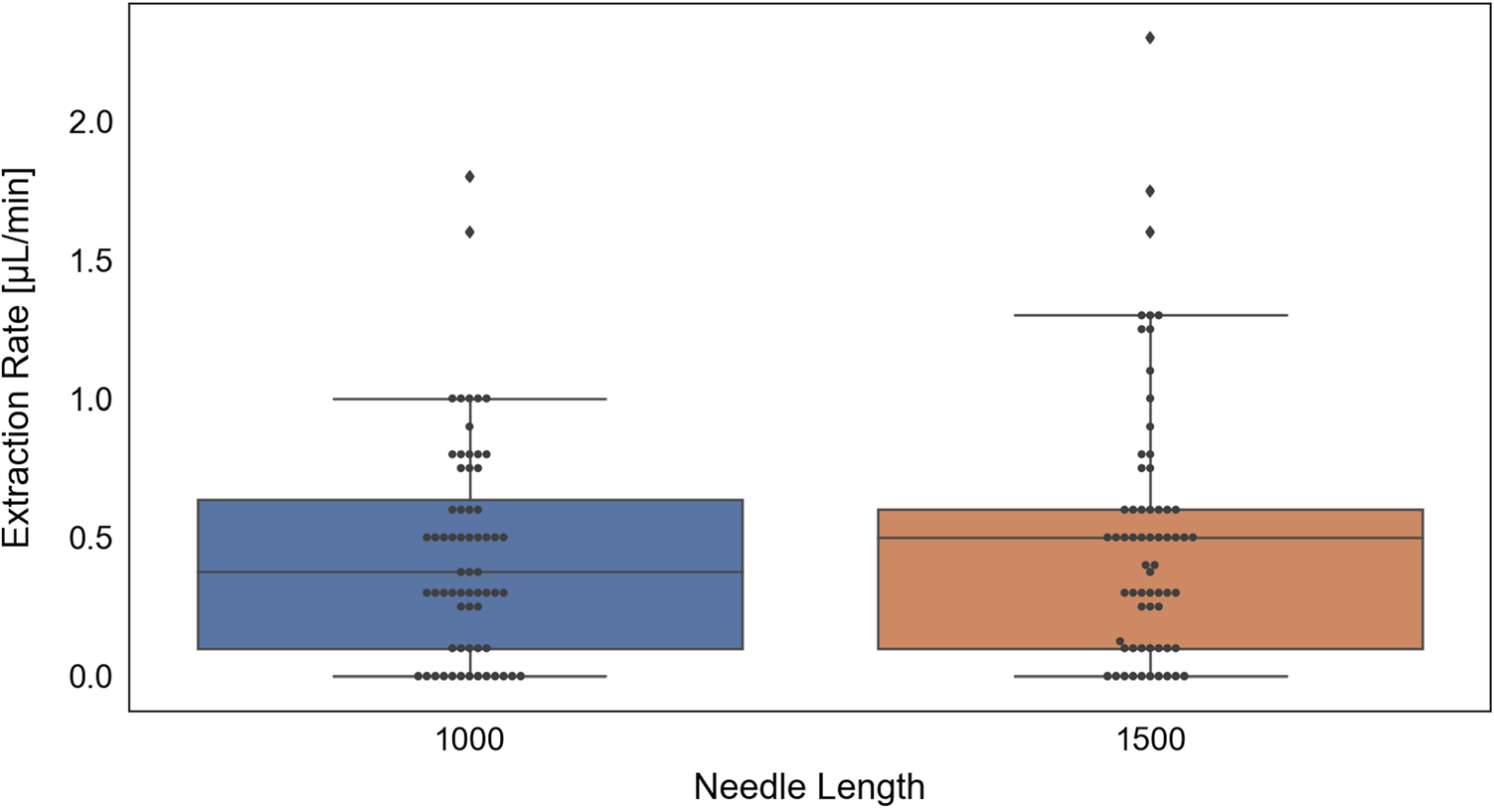
Comparison of MAs with 1000 μm versus 1500 μm needle lengths

We tested all 8 MA prototypes in CD Hairless rats and found that only the concave (CVE 1500) prototype had significantly better extraction rates, compared to the flat (FLT 1500) base prototype (*p* = 0.03). Extraction rates for each of the prototypes are detailed in Table 3 and Fig. 4. We did not measure any significant differences in extraction rates for the holders with 1000 μm versus 1500 μm needle lengths (Fig. 5). However, the different tip geometries were found to have varying effects on ISF extraction. When a sharp curvature like concave (Fig. 3c) is introduced in the design, it can accelerate fluid extraction measured in extraction volume per unit time (µl/min). This could be due to localized pressure differences around the needle. Alternately, the faster extraction rates measured in the concave models could be due to a compression effect, where the sides of the concave holder tip essentially act to push the skin down at the holder interface while lifting the skin in between the throat of the MN holder and, thus, slightly increasing needle penetration depth and/or localized pressure. Moreover, the concave (CVE) model, as shown in Figs. 2, 3a, pushes the skin inwards toward the needle, whereas the convex (CVX) model pushes the skin away from the needle. This could also account for both the increased extraction rate measured using the concave prototypes, as well as the decreased percent of total needles that also extracted blood in the concave models (Table 3).

**Table 3:** MA prototype extraction rates (*n* = 16 for each design)

Design ID	Mean extraction rate (µl/min)	Standard deviation (µl/min)	% Of total needles that also extracted blood (*n* = 80 total needles per group)
FLT 1000	0.41	0.35	11.3
FLT 1500	0.44	0.35	13.8
CVE 1000	0.71	0.51	2.5
CVE 1500	0.85	0.64	2.5
CVX 1000	0.28	0.32	12.5
CVX 1500	0.32	0.21	18.8
BVL 1000	0.37	0.44	8.8
BVL 1500	0.44	0.46	16.3

## Conclusions

Four different types of MAs were designed and 3D-printed, each with both 1000 μm and 1500 μm needle insertion depths. The four MN holder designs (flat, concave, convex, and beveled) all had different extraction rates, however, only the concave design had significantly increased ISF extraction rates, compared with the flat base model (p = 0.03). We did not measure any significant differences in extraction rates using 1000 μm versus 1500 μm needle insertion depths. Future studies measuring the local pressure differences between MA prototypes could further aid in the development of MA assemblies and patches for the successful extraction and analysis of ISF for biomedical and clinical applications. These results suggest that the specific geometry of the microneedle holder throat may be a critical factor in further optimizing interstitial fluid collection.
